# Feasibility and Efficacy of Adjuvant Chemotherapy With Gemcitabine After Liver Transplantation for Perihilar Cholangiocarcinoma - A Multi-Center, Randomized, Controlled Trial (pro-duct001)

**DOI:** 10.3389/fonc.2022.910871

**Published:** 2022-10-06

**Authors:** Moritz Schmelzle, Christian Benzing, Lutz Fischer, Uta Herden, Martina Sterneck, Utz Settmacher, Astrid Bauschke, Ulf Neumann, Uwe Pelzer, Tobias Müller, Christian Strassburg, Hauke Lang, Thomas Becker, Alfred Königsrainer, Silvio Nadalin, Markus Quante, Andreas Paul, Helmut Friess, Jürgen Klempnauer, Nicolas Richter, Florian Vondran, Andreas Pascher, Thomas Rösch, Wenzel Schöning, Felix Krenzien, Robert Öllinger, Daniel Seehofer, Peter Neuhaus, Johann Pratschke

**Affiliations:** ^1^ Department of Surgery, Charité – Universitätsmedizin, corporate member of Freie Universität Berlin, Humboldt-Universität zu Berlin, and Berlin Institute of Health, Berlin, Germany; ^2^ Department of General, Visceral, Thoracic and Transplant, University Hospital rechts der Isar, München, Germany; ^3^ Department of Visceral Transplant Surgery, University Hospital Hamburg-Eppendorf, Hamburg, Germany; ^4^ Department of Medicine I, University Medical Center Hamburg Eppendorf, Hamburg, Germany; ^5^ Department of General, Visceral and Vascular Surgery, University Hospital Jena, Jena, Germany; ^6^ Department of General, Visceral and Transplant Surgery, University Hospital RWTH Aachen, Aachen, Germany; ^7^ Department of Medicine - Hematology, Oncology and Tumour Immunology, Charité - Universitätsmedizin Berlin, Berlin, Germany; ^8^ Department of Medicine - Hepatology and Gastroenterology, Charité - Universitätsmedizin Berlin, Berlin, Germany; ^9^ Department of Medicine I, University Hospital Bonn, Bonn, Germany; ^10^ Department of General, Visceral and Transplant Surgery, University Hospital Mainz, Mainz, Germany; ^11^ Department of General, Visceral, Thoracic, Transplant and Pediatric Surgery, University Hospital Schleswig-Holstein, Kiel, Germany; ^12^ Department of General, Visceral and Transplant, University Hospital Tübingen, Tübingen, Germany; ^13^ Department of General, Visceral and Transplant, University Hospital Essen, Essen, Germany; ^14^ Department of General, Visceral and Transplant Surgery, Medizinische Hochschule Hannover, Hannover, Germany; ^15^ Department of General, Visceral and Transplant, University Hospital Münster, Münster, Germany; ^16^ University Hospital Hamburg-Eppendorf, Klinik und Poliklinik für Interdisziplinäre Endoskopie, Hamburg, Germany; ^17^ Department of Visceral, Transplant, Thoracic and Vascular Surgery, University Hospital Leipzig, Leipzig, Germany

**Keywords:** perihilar and intrahepatic cholangiocarcinoma, klatskin tumor, adjuvant chemotherapy, gemcitabine, liver transplantation, proximal bile duct cancer, biliary tract cancer

## Abstract

**Background:**

Liver transplantation (LT) is considered a therapeutic option for unresectable perihilar cholangiocarcinoma (PHC) within defined criteria. It remains uncertain whether patients can safely receive adjuvant chemotherapy after LT.

**Methods:**

We performed a prospective, multi-center, randomized, non-blinded two-arm trial (pro-duct001). Patients after LT for unresectable PHC within defined criteria were randomized to adjuvant gemcitabine (LT-Gem group) and LT alone (LT alone group). The primary objective was to investigate if adjuvant chemotherapy is feasible in ≥ 85% of patients after LT. The primary endpoint was the percentage of patients completing the 24 weeks course of adjuvant chemotherapy. Secondary endpoints included overall survival (OS) and disease-free (DFS), and complication rates.

**Results:**

Twelve patients underwent LT for PHC, of which six (50%) were eligible for randomization (LT-Gem: three patients, LT alone: three patients). Two out of three patients discontinued adjuvant chemotherapy after LT due to intolerance. The study was prematurely terminated due to slow enrollment. One patient with PHC had underlying primary sclerosing cholangitis (PSC). Tumor-free margins could be achieved in all patients. In both the LT-Gem and the LT alone group, the cumulative 1-, 3-, and 5-year OS and DFS rates were 100%, 100%, 67%, and 100%, 67% and 67%, respectively.

**Conclusions:**

This prospective, multi-center study was prematurely terminated due to slow enrollment and a statement on the defined endpoints cannot be made. Nevertheless, long-term survival data are consistent with available retrospective data and confirm defined criteria for LT. Since more evidence of LT per se in unresectable PHC is urgently needed, a prospective, non-randomized follow-up study (pro-duct002) has since been launched.

## Introduction

Major hepatectomy is the only established treatment option with potentially curative intent for perihilar cholangiocarcinoma (PHC) (1–5). Over the last years, several surgical strategies and tailored approaches have been defined and good long-term survival can be achieved in resectable tumors (1, 6). However, certain constellations can make resection impossible even in the case of “locally resectable” findings, such as primary sclerosing cholangitis (PSC) or liver cirrhosis, potentially being associated with impaired liver functions (1, 7). An increasing number of retrospective analyses from Europe and the United States (US) proposes liver transplantation (LT) as an alternative in some of those patients (8–10).

The idea of LT for biliary tract cancer, with or without removing the entire bile duct system by hepatoduodenopancreatectomy (HDP), dates back to the early 1990s (11). The rate of potentially curative surgery in PHC was convincing after LT, however, this was not reflected in improved long-term survival compared to extended hepatectomy, even after HPD (4, 12, 13). In this respect, LT must be well justified against liver resection in PHC, if only because of the dramatic organ shortage. At present, there is a broad consensus that LT represents a good alternative for non-resectable tumors within well-defined criteria and should be offered as a “rescue option” to a small group of patients (8).

Beyond the question of LT *per se* as a sensible treatment option in PHC, there is an urgent need to achieve more evidence for perioperative concepts in transplant oncology. Neoadjuvant radiochemotherapy (RCTx) before LT for PHC is not common in much of Europe, especially as its value has not been confirmed in controlled trials (8, 10). The question of an adjuvant offer after LT, however, is more topical than ever after the positive results of the *BILCAP* trial (14) and in view of current achievements in antibody-based immunotherapy (15). It seems all the more interesting to clarify how many of the transplanted PHC patients qualify for adjuvant therapy.

We here report on the results of the *pro-duct001* study. The purpose of this trial was to transplant patients with unresectable and well-defined PHC and then randomize them to an adjuvant chemotherapy arm and an observation arm. The primary objective of the study was to determine if a clinically meaningful percentage of patients could receive adjuvant chemotherapy after LT. Secondary objectives were to obtain preliminary data on survival rates after LT and either adjuvant or no adjuvant chemotherapy for PHC using strict selection criteria. Unfortunately, due to recruitment difficulties, the study had to be terminated prematurely.

## Methods

### Trial Design

The *pro-duct001* study was an investigator-initiated, multicenter, randomized, controlled trial, which recruited patients with unresectable PHC in 12 German transplant centers. It was intended to offer those patients the possibility to undergo LT within a controlled trial; the envisaged sample size was n = 60 patients. The study was designed as a pilot study to create data on LT for PHC using strict patient selection either with or without adjuvant chemotherapy and to evaluate if the obtained survival rates justify a subsequent larger trial using the same treatment algorithm.

The trial protocol was approved by the German competent authority (BfArM, Federal Institute for Drugs and Medical Devices; German: Bundesinstitut für Arzneimittel und Medizinprodukte) and by the leading ethics committee at Charité - Universitätsmedizin Berlin (DRKS00000805, 61-3910-4036526). The trial design followed the CONSORT guidelines and complied with the principles of the declaration of Helsinki from 1975 (Version Sommerset West 1996) (16), as well as all pertinent national laws and the ICH guidelines for Good Clinical Practice (GCP) issued in June 1996 and CPMP/ICH/135/95 from September 1997.

### Study Population

Patients with PHC aged 18 to 65 years within defined criteria (see below) were eligible for the trial. All patients were informed of the nature of the study and provided written informed consent (according to AMG §40 (1) 3b).

### Listing for Liver Transplantation

All patients underwent Endoscopic Retrograde Cholangiography (ERC) with brush cytology using standard operative procedures (SOP) or tumor biopsy (*via* ERC only). If all protocol criteria for the diagnosis of PHC were given and metastatic disease was excluded by staging investigations, all patients underwent staging laparotomy with lymph node retrieval (using defined SOP) before listing for LT. This was to exclude lymph node metastases, peritoneal carcinomatosis or other intraabdominal metastatic manifestations.

In patients without PSC, clinical diagnosis of PHC was based on ERC plus a second method (computed tomography (CT) or magnetic resonance imaging (MRI)). Cytology was obtained during ERC, but a cytological result of carcinoma or severe dysplasia was not mandatory. In patients with PSC, histological diagnosis of cholangiocarcinoma (obtained *via* ERC) was mandatory or evidence of dominant stenosis plus cytological diagnosis of severe dysplasia or two subsequent cytological results of severe dysplasia or carcinoma, whereby the second has been obtained after two weeks of antibiotic treatment to exclude inflammatory changes.

Staging laparotomy with diagnostic hilar lymphadenectomy (at least 2 lymph nodes) before priority listing was mandatory in all patients. Lymphadenectomy was performed at the hepatoduodenal ligament (common hepatic artery and portal vein) and the upper pancreatic margin. The perihilar region was left untouched for oncological reasons. The tumor had to be judged as not curatively resectable by an experienced hepatobiliary surgeon (> 50 liver resections for PHC). An on-line review of defined patient data and acceptance for priority listing by a Eurotransplant expert panel consisting of two experts recruited from the Eurotransplant Liver Allocation Committee was a prerequisite for listing. LT was intended within 3 months after listing and a match model of end-stage liver disease (matchMELD) of 38 increasing to 40 was assigned after one month. LT was planned with extrahepatic bile duct resection and regional lymphadenectomy according to the SOP.

### Contraindications for Liver Transplantation

Besides general contraindications, LT was contraindicated in tumors larger than 3 cm in diameter (as determined by a visible tumor mass on CT or MRI), in tumors infiltrating adjacent organs or the main trunk of the hepatic artery and/or in tumors with known lymph node or distant metastasis (as determined by CT scan and laparotomy). Further contraindications for LT were a highly elevated Carbohydrate Antigen 19-9 (CA 19-9) levels (> 1000 U/ml) and tumors being suspicious for gallbladder cancer.

Patients after previous or intended photodynamic therapy, radiation, chemotherapy, brachytherapy or combinations of these procedures were excluded for LT. Further contraindications for LT were previous tumor biopsy (except *via* ERC), systematic lymphadenectomy (except SOP defined staging laparotomy), surgical preparation at the region of the hepatoduodenal ligament (except cholecystectomy for other reasons) or previously completed or attempted surgery for PHC.

### Inclusion Criteria for Randomization

Inclusion criteria for randomization were LT within three months after listing and the possibility to start adjuvant chemotherapy not later than 10 weeks after LT. Histologically proven PHC and curative resection of the tumor (R0) was mandatory for study inclusion. Patients were stratified in the treatment groups in case of previously unknown hilar lymph node metastases nearby the tumor region in the final pathological report. The complete list of inclusion and exclusion criteria is included in the supplementary material (**Supplementary Table S1**).

**Figure 1:**
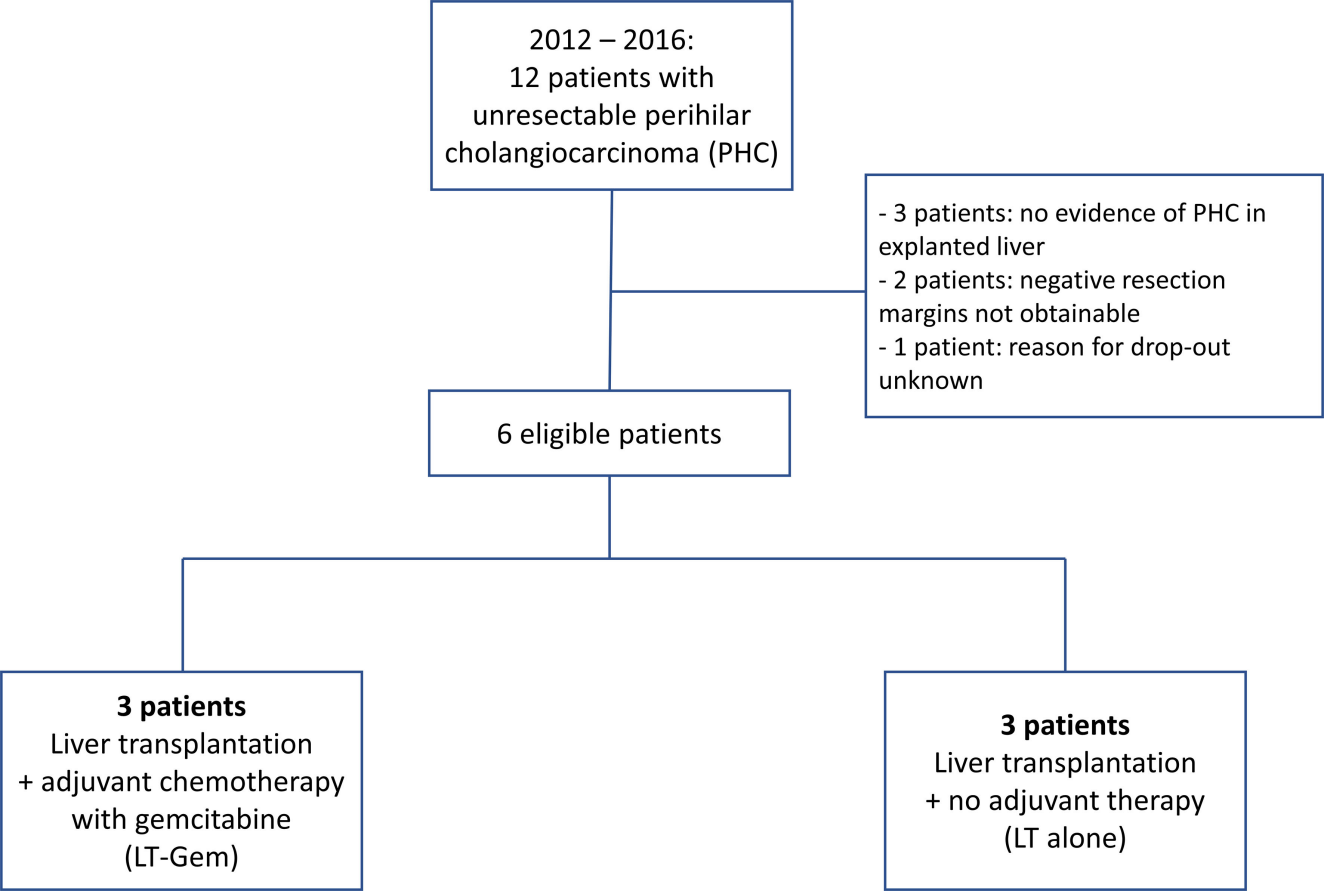
CONSORT flow chart of patients.

Patients were scheduled for a total of 24 weeks of adjuvant chemotherapy with gemcitabine monotherapy. The standard adjuvant chemotherapy after resection of PHC is gemcitabine with cisplatin (gem-cis) (17). However, gem-cis combination therapy is known to increase the probability of chemotherapy-associated toxicity such as neutropenia which needed to be taken into account since it was administered to highly immunosuppressed patients (17). The chemotherapy comprised 6 cycles of 28 days each. Gemcitabine was administered on days 1 and 8 and 15 in each cycle. The starting dose was 800 mg/m2 during the first two cycles to reduce toxicity in the early post-transplant period. If tolerated the dosage during cycles 3 to 6 was increased to 1000 mg/m2 per application. Patients in the control group did neither receive adjuvant treatment nor placebo. **Figure 1** shows an overview of inclusion criteria.

### Endpoints

The primary objective was to investigate if adjuvant chemotherapy with gemcitabine is feasible in ≥ 85% of patients after LT. Secondary objectives were to obtain preliminary data on recurrence- and survival rates after LT for PHC using strict selection criteria and either adjuvant or no adjuvant chemotherapy. The primary endpoint was the percentage of patients completing the 24 weeks course of adjuvant chemotherapy. Secondary endpoints were disease-free survival (DFS) at 12 months, overall survival (OS) at 3 and 5 years, complication rate (protocol defined high-grade toxicities grade 3/4) and number of patients, which could not be randomized (due to perioperative complications, no detectable bile duct cancer in the explanted liver or other reasons).

## Results

### Baseline Characteristics

From July 2012 to September 2016, 12 patients from four German transplant centers were screened. All 12 patients subsequently underwent staging laparotomy. LT was performed in 10 of 12 patients (83%). In two patients (17%), LT was not performed due to tumor infiltration of the common hepatic artery and peritoneal carcinomatosis, respectively. Another four of the remaining 10 patients (40%) could not be randomized due to missing histological tumor proof in three patients, and due to pending formalities in one patient.

Median age was 61 years (41-63), median time between the date of diagnosis and listing and the date of diagnosis and LT were 18 (4-25) days and 73 (42-81) days, respectively. One patient (17%) had underlying PSC and ulcerative colitis. Four patients (67%) experienced postoperative complications after LT, which could all be managed conservatively [according to Clavien/Dindo grade 1 and 2 (18)]. **Table 1** provides an overview of all baseline characteristics.

**Table 1:** Baseline characteristics.

	All patients n = 6	LT-Gem n = 3	LT alone n = 3
Age (years)^1^	60.5 (41-63)	61 (52-63)	60 (41-62)
Body mass index (kg/m²)^1^	25.5 (21-32)	29.5 (21-32)	25 (24-26)
Time from diagnosis to listing (days)^1^	17.5 (4-25)	21 (14-25)	11 (4-22)
Time from diagnosis to transplantation (days)^1^	73 (42-81)	62 (42-78)	73 (73-81)
Gender^2^			
Male	5 (83)	3 (100)	2 (67)
Female	1 (17)	0	1 (33)
Medical history^2^			
Arterial hypertension	3 (50)	2 (67)	1 (33)
Coronary heart disease	3 (50)	2 (67)	1 (33)
Primary sclerosing cholangitis	1 (17)	0	1 (33)
Ulcerative colitis	1 (17)	0	1 (33)
Postoperative complications according to Clavien/Dindo Classification^2^			
Grade 0	0	0	0
Grade I	0	0	0
Grade II	5 (83)	3 (100)	2 (67)
Grade IIIa	0	0	0
Grade IIIb	0	0	0
Grade IVa	1 (17)	0	1 (17)
Grade IVb	0	0	0
Grade V	0	0	0
Carbohydrate antigen 19-9 (kU/l)^1^	40.5 (9.0-165.4)	54.9 (13.0-136.0)	26 (9.0-165.4)
Preoperative bilirubin (mg/dl)^1^	1.3 (0.3-11.2)	0.7 (0.3-11.2)	1.8 (0.6-1,9)
Preoperative creatinine (mg/dl)^1^	0.8 (0.4-1.0)	0.7 (0.6-0.8)	0.8 (0.4-1.0)
Preoperative platelet count (10^9^/liter)^1^	232.5 (170-422)	188 (170-195)	300 (270-422)
Preoperative international normalized ratio^1^	1.1 (0.8-1.2)	1.0 (0.8-1.2)	1.1 (1.0-1.1)

^1^Presented as median and range,^2^Presented as count and proportions (%).

With regards to histopathological findings, tumor-free resection margins were obtained in all patients. Most resected tumors were moderately differentiated (G2, 4 of 6, 67%) and lymph-node negative (4 of 6, 67%, **Table 2**). The immunosuppressive regimen consisted of tacrolimus + mycophenolate mofetil (MMF, 4 patients, 67%), cyclosporin A + MMF (one patient, 17%), and tacrolimus alone (one patient, 17%), respectively. All patients received corticosteroids (**Table 2**).

**Table 2:** Immunosuppressive regimen and histopathological findings.

No.	Group	Immuno-suppression	Cortico-steroids	Others	Chemotherapygiven	pT	G	Pn	V	N	R	Lymph nodes at SL	Lymph Nodes at LT	Lymph Nodes positive
1	LT-Gem	tacrolimus	yes	MMF	yes	pT2	G2	Pn0	V0	N0	R0	5	12	0
2	LT alone	tacrolimus	yes	MMF	no	pT2	G1	Pn1	V0	N0	R0	3	11	0
3	LT alone	tacrolimus	yes	MMF	no	pT2	G3	Pn0	V0	N0	R0	5	9	0
4	LT alone	cyclosporin A	yes	MMF	no	pT2	G2	Pn1	V0	N1	R0	1	8	1
5	LT-Gem	tacrolimus	yes	MMF	yes	pT2	G2	Pn1	V0	N0	R0	3	8	0
6	LT-Gem	tacrolimus	yes	none	yes	pT2	G2	Pn1	V0	N1	R0	3	6	3

No., Patient number; LT-Gem, liver transplantation and adjuvant gemcitabine (intervention group); LT alone, liver transplantation alone (control group); MMF, mycophenolate mofetil; SL, staging laparotomy; LT, liver transplantation.

### Donor Data

Median donor age was 55.5 (21-73) years. Median cold ischemia time was 510 (307-710) minutes. Cause of death was intracranial bleeding in 2 donors (33%) as well as hypoxic brain damage (n=2, 33%), stroke in one patient (17%). In one patient cause of death was not documented. With regards to cytomegaly virus (CMV) status, 4 of 6 (67%) donors were CMV negative.

### Patients’ Outcome

In the LT-Gem group, Gemcitabine could be administered per protocol in one patient (33%) and was discontinued in the remaining two patients (66%) due to intolerance (deterioration of liver transplant function and radiological evidence of pneumonitis, respectively) after 1.5 and 2.5 months, respectively. Since only three patients received adjuvant chemotherapy, the statistical power was too low to analyze the primary endpoint.

### Secondary Endpoints

In both the LT-Gem and the LT alone group, the cumulative 1-, 3-, and 5-year OS and DFS rates were 100%, 100%, 67%, and 100%, 67% and 67%, respectively. One patient (33%) from the LT-Gem group had peritoneal recurrence after 36 months and died after 39 months. One patient (33%) from the LT alone group had peritoneal recurrence after 36 months and died after 38 months.

## Discussion

We here report on the first prospective randomized trial on LT for unresectable PHC within defined criteria. It was hoped to obtain robust data on LT for PHC in combination with adjuvant chemotherapy to justify a larger trial using the same treatment algorithm. Unfortunately, due to recruitment difficulties, the study had to be terminated prematurely and no final conclusions on the objectives are possible. This fact, alone, however, does not seem to be without significance. Obviously, the constellation necessary for LT is very rarely present in PHC, given the strict selection criteria and the centers’ high expertise in complex hepatobiliary surgery. A small number of PHC patients qualifying for LT is desirable in view of the organ shortage. With regard to the design and planning of future studies, however, the possibility of being able to conduct prospective randomized trials in a meaningful way must be assessed as very critical. Based on our experiences, it will likely not be possible within the current framework to substantiate the significance of LT for PHC and associated questions, such as perioperative concepts.

Given the favorable survival after LT in our series, the selection criteria appear to be very predictive from an oncological point of view and to be suitable for identifying a subset of patients that really benefits from LT. It would be interesting to learn to what extent these criteria could probably be extended in the future, e.g. with regard to the size of the tumor. This would be an important aspect, insofar as tumors up to 3 cm are usually resectable anyways (19). The primary objective of the study was to determine the percentage of patients who are able to receive adjuvant chemotherapy after LT. No final conclusions can be drawn in this regard, although the fact that two out of three patients were unable to complete chemotherapy does not give confidence. It remains unclear whether this is even necessary after LT for PHC from an oncological point of view within these strict selection criteria (14). Especially for very early PHC, LT might well be sufficient alone as a most radical treatment option. This would again confirm the significance of wide resection margins, as postulated by Neuhaus et al. in the early 1990s (2).

There are other interesting aspects to draw from this study, although they have to be interpreted cautiously due to the small number of patients. Survival rates of 67% confirm previous retrospective data from European and US transplant centers. Mantel et al. reported a 5-year OS of 59% after LT in 28 PHC patients identified using the European Liver Transplant Registry (ELTR) to fulfill the Mayo criteria (10). Interestingly, none of those ELTR patients had undergone neoadjuvant RCTx, which corresponds to the pro-duct001 study protocol. As those data are comparable to LT data from the US, achieved after the use of the neoadjuvant Mayo Clinic neoadjuvant RCTx protocol, survival rates might well be attributed to strict selection alone. Further, RCTx was often used to justify the high rate of patients without microscopic tumor detection in the final histological examination after LT. Of note, there was a high proportion of histologically unconfirmed tumors in the explant liver in the pro-duct001 study, despite the fact that patients had no neoadjuvant radiochemotherapy (RCTx). In this respect, we assume that there is still a considerably large discrepancy between the preoperatively suspected diagnosis and histological confirmation postoperatively. There are currently emerging and still experimental approaches being developed. These techniques, such as circulating cell-free deoxyribonucleic acid (cfDNA) may help to increase the diagnostic accuracy in the future (20).

Another interesting aspect of the pro-duct001 study is the considerably high rate of transplanted patients with evidence of lymph node metastases (33%) in the final histology. This is even more astonishing given the high adherence to the SOP for diagnostic hilar lymphadenectomy. The rate of postoperatively confirmed positive lymph nodes is lower in some publications, with histological data being not reported in others (9, 21). Whether this discrepancy can, at least in parts, be explained by a downstaging effect of the neoadjuvant therapy in other series remains uncertain (9, 21). It must further be critically questioned whether a number of lymph nodes should be mandatory in order to be able to reliably exclude a lymph node metastasis before LT. Given the fact that both lymph node positive patients in the pro-duct001 trial are still disease-free after five years of surveillance, however, we certainly need to better assess the prognostic value of positive lymph nodes. Even though there are far too few patients to make a valid assessment in this regard, it is conceivable that patients with a proportion of positive lymph nodes ≤ 30%, as seen in those two patients (1/10 and 3/9), might also benefit from LT (1).

The overall evidence on side effects after systemic anti-cancer treatment (SACT) is low. However, there are some reports that show that chemotherapy for colorectal liver metastasis is generally well tolerated after LT. Adverse events (AE) do not appear to be more frequent when compared to patients without history of LT (22). Adjuvant systemic therapy after LT for hepatocellular carcinoma (HCC) appears to be well-tolerated as well although the authors focused on efficacy outcomes rather than on safety (23). Another study evaluated LT recipients who developed different malignancies (mainly skin cancer). The authors report that outcomes were poor with high grades of dose-limiting toxicities (24). The results of these small series have to be interpreted with caution. The patient cohorts are heterogenous with different malignancies and various chemotherapy agents. Also, there are other factors such as general conditions and age that are not systematically taken into account. Nevertheless, it can be stated that the application of SACT in this sensitive patient group is not easy in principle. In other studies, such as the SECA 1 and 2 trials (25, 26), no adjuvant chemotherapy was applied after LT for colorectal liver metastases (CRLM). There are considerations to shift the timing of the chemotherapy into the preoperative period as neoadjuvant treatment which could contribute to limiting the toxicities in the post-transplant period. First data on neoadjuvant chemotherapy in patients with intrahepatic cholangiocarcinoma show promising results (27).

The pro-duct001 trial suggested that adjuvant chemotherapy might not necessarily increase survival after LT in early PHC. Furthermore, we noted only a very small percentage of eligible patients with early PHC fulfilling the Mayo-criteria that are considered not resectable at experienced centers. Therefore, the pro-duct002 protocol has been simplified aiming to provide more data on LT for PHC in a prospective setting rather than to randomize patients after LT to adjuvant chemotherapy or observation.

In summary, controlled studies are currently not meaningfully possible and robust statements cannot be expected in this field. We are therefore obliged, for ethical reasons alone, to continue to offer LT for PHC only in the context of studies, which incidentally also corresponds to directives in Germany (28). In Germany we have designed a prospective, multi-center, non-randomized, non-blinded single-arm trial [Microscopic Tumor Clearance after Liver Transplantation for Proximal Bile Duct Cancer (pro-duct002) trial (DRKS00013276)). This allows us to offer PHC patients, who are not or not likely curatively resectable, the possibility to undergo LT within a controlled trial in view of the otherwise unfavorable prognosis. The primary objective of the study is to achieve more data on LT for PHC using established selection criteria, especially on the microscopic tumor clearance and recurrence rates after LT. The pro-duct002 study is currently recruiting participants.

## Conclusions

We must learn our lessons from this study and conclude that prospective randomized trials are difficult to implement due to the rarity of this tumor and the strict inclusion criteria for LT. Excellent long-term results of LT for PHC from retrospective analyses could be confirmed, which underlines the value of LT in a well-defined subset of patients with unresectable PHC. In this respect, patients should be referred to centers that have both a high level of expertise in the complex surgery of PHC and also offer patients the option of LT. Ideally, these centers should participate in prospective studies and contribute to the data acquisition of these rare tumors.

## Data Availability Statement

The raw data supporting the conclusions of this article will be made available by the authors on reasonable request.

## Ethics Statement

The studies involving human participants were reviewed and approved by Ethikkommission der Charité - Universitätsmedizin Charitéplatz 1 10117 Berlin Germany. The patients/participants provided their written informed consent to participate in this study.

## Author Contributions

All authors listed have made a substantial, direct and intellectual contribution to the work, and approved it for publication. All authors contributed to the article and approved the submitted version.

## Funding

This study was funded by Astellas Pharma GmbH.

## Conflict of Interest

Authors MS, CB, WS, FK, RO, PN and JP are corporate members of Freie Universität Berlin.

The authors declare that this study received funding from Astellas Pharma GmbH. The funder was not involved in the study design, collection, analysis, interpretation of data, the writing of this article or the decision to submit it for publication.

## Publisher’s Note

All claims expressed in this article are solely those of the authors and do not necessarily represent those of their affiliated organizations, or those of the publisher, the editors and the reviewers. Any product that may be evaluated in this article, or claim that may be made by its manufacturer, is not guaranteed or endorsed by the publisher.
