# Supplementary material for: Feasibility and Efficacy of Adjuvant Chemotherapy With Gemcitabine After Liver Transplantation for Perihilar Cholangiocarcinoma - A Multi-Center, Randomized, Controlled Trial (pro-duct001)
Source: Front Oncol. 2022 Oct 6;12:910871. doi: 10.3389/fonc.2022.910871 (PMC9624225; doi:10.3389/fonc.2022.910871)
Supplement: Supplementary file 1 [file Table_1.docx]

**Supplementary Tables**

**Supplementary Table S1: Complete list of inclusion and exclusion criteria**

| **Study population** | **Patients after liver transplantation for hilar cholangiocarcinoma** |
| --- | --- |
| **Listing for liver transplantation** | - diagnosis of cholangiocarcinoma in patients with primary sclerosing cholangitis (PSC): i.e. histological diagnosis of cholangiocarcinoma (obtained via ERC) or dominant stenosis plus cytological diagnosis of severe dysplasia or two subsequent cytological results of severe dysplasia or carcinoma whereby the second has been obtained after 2 weeks of antibiotic treatment to exclude inflammatory changes  - diagnosis of hilar cholangiocarcinoma in patients with Klatskin tumors without PSC: clinical diagnosis of proximal bile duct cancer based on ERC plus a second method (CT or MRI), cytology is obtained during ERC, but a cytological result of carcinoma or severe dysplasia is not mandatory  - tumor not curatively resectable as judged by an experienced hepatobiliary surgeon (> 50 liver resections for hilar cholangiocarcinoma)  - on-line review of defined patient data and acceptance for priority listing by an Eurotransplant expert panel consisting of two experts recruited from the Eurotransplant Liver Allocation Committee; in case of split decision addition of a third expert for definite decision on priority listing with a respective matchMELD  - obligatory staging laparotomy before priority listing (see SOP staging laparotomy)  - age between 18 and 65 years  - negative pregnancy test  - informed consent before study specific procedures are performed (i.e. at the time of randomization for chemotherapy, all other procedures are clinical routine procedures in the management of these patients) |
| **Contraindications for liver transplantation:** | - locally very advanced, unresectable tumor infiltrating adjacent other organs, the main trunk of the hepatic artery  - a visible tumor mass on CT or MRI scan larger than 3 cm in diameter  - highly elevated CA 19-9 levels (> 1000 U/ml)  - tumors suspicious for gallbladder cancer  - known lymph node or distant metastasis (determined mandatory by CT scan and laparotomy, further investigations if deemed necessary, a PET scan is recommended)  - patients undergoing multi-organ transplantation or have undergone previous solid organ or bone marrow transplantation  - previous or intended photodynamic therapy, radiation, chemotherapy, brachytherapy or combinations of these procedures  - previous tumor biopsy (except via ERC) systematic lymphadenectomy (except SOP defined staging laparotomy), surgical preparation at the region of the hepatoduodenal ligament (except cholecystectomy for other reasons) or previous completed or attempted surgery for hilar cholangiocarcinoma  - patients with known hypersensibility for gemcitabine  - pregnancy or breastfeeding  - patients unwilling to consent to saving and propagation of pseudonymized medical data for study reasons  - general contraindications for liver transplantation  - subjects who are legally detained in an official institute  - HIV infection (based on testing performed during the transplant-evaluation or within 6 months before) |
| **Inclusion criteria for randomization (adjuvant chemotherapy versus observation)** | - liver transplantation within three months after listing for liver transplantation  - histologically proven proximal bile duct cancer  - curative resection of the tumor (R0)  - in case of previously unknown hilar lymph node metastases nearby the tumor region in the final pathological report: stratification in the treatment groups  - no previous photodynamic therapy, radiation, chemotherapy, brachytherapy or combinations of these procedures  - CT scan of the chest and abdomen prior to randomization showing no detectable distant metastases  - possibility to start adjuvant chemotherapy between 4 and 8 weeks (but not later than 10 weeks) after liver transplantation  - sufficient bone marrow function (WBC > 3.5/nl, platelets > 80/nl, hemoglobin > 8 g/dl)  - sufficient renal function (creatinine clearance ≥ 30 ml/min estimated by the Cockcroft-Gault formula)  - sufficient liver function (bilirubin < 3 mg/dl)  - if female either be postmenopausal, surgically sterile or have a negative pregnancy test as part of the screening investigations. Women of childbearing potential also must agree to use an acceptable highly effective contraception method of birth control (defined as pearl index < 1), if necessary also for partners of test persons or complete abstinence of intercourse (from the date of liver transplantation until 2 months after end of adjuvant chemotherapy). if male must agree to use an acceptable method of birth control, as determined by the investigator, from the date of liver transplantation until 2 months after end of adjuvant chemotherapy  - all patients must be informed of the nature of the study and provide written informed consent before any study specific procedures are performed (according AMG §40 (1) 3b) |
| **Exclusion criteria for randomization (adjuvant chemotherapy versus observation)** | - patients with biliary sepsis, intrahepatic abscesses or other complications (e.g. severe liver dysfunction) contraindicating adjuvant chemotherapy  - contraindications against study medication (including auxiliary substances)  - pregnancy or breastfeeding  - induction therapy with antibodies other than IL-2 receptor antibodies as primary immunosuppression after liver transplantation (induction therapy with IL-2 receptor antibodies is allowed, anti-rejection treatment using antibodies is not a contraindication)  - therapy with mTOR-inhibitors (sirolimus, everolimus)  - any clinically significant medical or surgical condition, that in the investigator‘s opinion would preclude adjuvant chemotherapy  - participation in other clinical trials |
